# Isolated choroidal melanocytosis: clinical update on 37 cases

**DOI:** 10.1007/s00417-020-04919-x

**Published:** 2020-09-09

**Authors:** James J. Augsburger, Cassandra C. Brooks, Zelia M. Correa

**Affiliations:** 1 Department of Ophthalmology, University of Cincinnati College of Medicine, Medical Science Building, Room 5306, Cincinnati, OH 45267-0527 USA; 2 Department of Ophthalmology, Duke University School of Medicine, Durham, NC USA; 3 Wilmer Eye Institute, Johns Hopkins University School of Medicine, Baltimore, MD USA

**Keywords:** Ocular melanocytosis, Choroidal melanocytosis, Choroidal malignant melanoma

## Abstract

**Purpose:**

Isolated choroidal melanocytosis is a congenital melanocytic hyperpigmentation involving the choroid that is not associated with iridic or scleral features of ocular melanocytosis. The purpose of this work was to describe the clinical features and course of a relatively large series of patients with this disorder.

**Methods:**

A retrospective clinical study of 37 patients with isolated choroidal melanocytosis encountered in a single practice 1986–2018 was done. All lesions were 5 mm or larger in the largest basal diameter, homogeneously melanotic, and completely flat by conventional ocular ultrasonography.

**Results:**

The 37 patients ranged in age from 2 weeks to 87 years (mean 31.5 years, median 18 years) at initial diagnosis of the melanotic choroidal lesion. Arc length largest basal diameter of the melanotic choroidal lesion ranged from 5.5 to 37 mm (mean 14.6 mm, median 13 mm). The lesion extended beneath the fovea in 18 eyes and to the optic disc margin in 6 eyes. Ten of the lesions straddled the ocular equator, but the center point of all of the lesions was posterior to the equator. The retina was fully attached and appeared normal over the melanotic choroidal lesion in each of these eyes. None of the melanotic choroidal lesions exhibited clumps of orange pigment or drusen on its surface. The lesion was unilateral and unifocal in 36 of the 37 patients. One patient had bilateral choroidal melanocytosis that was isolated in one eye but associated with partial iris melanocytosis in the fellow eye. Three adult patients had a choroidal melanoma localized to the patch of choroidal melanocytosis at baseline. One other adult patient had a choroidal melanoma in the fellow eye at baseline. One pediatric patient had viable unilateral non-familial retinoblastoma in the fellow eye and two adult patients had a classic choroidal nevus in the fellow eye. None of the flat patches of choroidal melanocytosis that were monitored periodically after initial diagnosis expanded appreciably during follow-up ranging from 4.9 months to 15.2 years (mean 5.0 years, median 2.3 years).

**Conclusions:**

Isolated choroidal melanocytosis is a distinct clinical entity that must be distinguished from broad-based choroidal nevus, choroidal melanocytoma, small choroidal malignant melanoma, acquired bilateral patchy-streaky choroidal melanocytic fundopathy associated with disorders such as cutaneous vitiligo and Waardenburg syndrome, acquired bilateral zonal choroidal melanocytic fundopathy, and diffuse uveal melanocytic proliferation associated with systemic cancer. This disorder appears to predispose affected eyes to development of choroidal melanoma arising from the hypermelanotic patch.

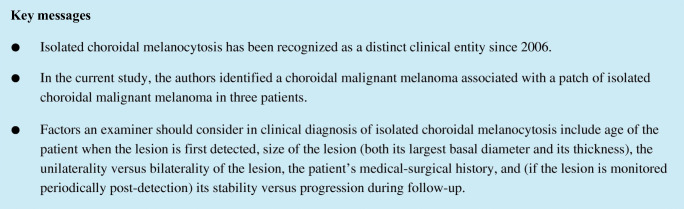

## Introduction

Isolated choroidal melanocytosis is a partial form of congenital ocular melanocytosis in which clinically evident melanotic hyperpigmentation of the eye is limited to the choroid. First described as a distinct clinical entity in 2006 [1], several other cases categorized as this disorder have been reported [2–8]. Isolated choroidal melanocytosis has the following clinical features: (1) a discrete patch of clinically flat choroidal hyperpigmentation that is indistinguishable from patches of choroidal involvement in persons with partial (sector) ocular melanocytosis [9]; (2) absence of ipsilateral iridic or anterior scleral melanocytosis; and (3) no measurable thickness of the melanotic choroidal lesion by B-scan ocular ultrasonography.

The purposes of this study were to describe an expanded group of patients with this disorder and report three patients who had a choroidal malignant melanoma that appeared to have arisen from the hypermelanotic choroidal patch.

## Patients and methods

The authors performed a retrospective descriptive study of patients with isolated choroidal melanocytosis encountered in their clinical practice during the period 1986 through 2018. Patients were identified by search of the practice’s patient name—clinical diagnosis database for cases coded as ocular melanocytosis, choroidal melanocytosis, or diffuse choroidal nevus. The medical records of all of these patients were reviewed individually. The inclusion criterion used to identify cases for this study was a patch of confluent melanotic choroidal hyperpigmentation consistent with congenital choroidal melanocytosis in one or both eyes. Exclusion criteria used to eliminate cases were (1) largest arc length basal diameter of the melanotic choroidal lesion less than 5 mm, (2) measurable thickness of the melanotic choroidal lesion greater than that of the surrounding uninvolved choroid by B-scan ultrasonography, (3) presence of ipsilateral anterior scleral or iridic features of ocular melanocytosis [9, 10], (4) acquired bilateral patchy-streaky choroidal melanocytic fundopathy consistent with the fundopathy of cutaneous vitiligo [11, 12] and Waardenburg syndrome [13–15], and (5) acquired bilateral zonal choroidal melanocytic fundopathy that is age-related or drug-induced [16, 17]. Lesion diameter less than 5 mm was used to exclude typical choroidal nevi, 95% of which have been reported to be smaller than this [18]. Measureable thickness of the lesion greater than the surrounding uninvolved choroid was used to exclude most choroidal nevi larger than 5 mm in basal diameter (most of which are measurably thicker than the surrounding choroid by ultrasonography [19]), choroidal melanocytomas, and small choroidal melanomas. None of the lesions in this series was evaluated by optical coherence tomography.

We abstracted demographic and medical-surgical historical information about each identified patient and eye-specific information (eye involved; best corrected visual acuity; arc length largest and smallest basal diameters of the melanotic choroidal lesion; location of the lesion in the fundus relative to the fovea, optic disc, and ocular equator; shape of the choroidal lesion (coded as either circular-ovoid or irregular-geographic); features of the margins of each lesion (coded as smooth or striated-feathered); absence versus presence of orange pigment (lipofuscin) clumps, drusen, and disruption and/or clumping of the retinal pigment epithelium overlying the lesion; absence versus presence of associated serous subretinal fluid overlying or surrounding the lesion; and absence versus presence of lesions categorized as choroidal nevus, choroidal malignant melanoma, or focal aggregates of normal or near-normal uveal melanocytes (FANNUMs) in the choroid [20]) about each affected and fellow eye from our office records. We created a comprehensive computerized database (SPSS for Windows) of this information and computed conventional descriptive statistics on the study cases.

## Results

Thirty-seven patients with isolated choroidal melanocytosis were identified by our records search (Table 1). This series included the 11 cases reported in our 2006 publication on this topic [1]. These cases are identified by their case numbers in the 2006 article in Table 1. Thirty-six of these cases were coded correctly (by either JJA or ZMC) as isolated choroidal melanocytosis at the time of their initial evaluation in the Ocular Oncology Service. One case (case 32 in Table 1) was coded incorrectly initially (by JJA) as diffuse choroidal nevus in 1991. The correct revised diagnosis of isolated choroidal melanocytosis was made during a retrospective chart review in 2018. No case coded as complete generalized or partial (sectoral) ocular or oculodermal melanocytosis underwent recoding as isolated choroidal melanocytosis during retrospective chart review.

**Table 1:** Raw data on 37 cases of isolated choroidal melanocytosis. Cases are listed in ascending order by age at initial evaluation in the Ocular Oncology Service. This series includes 11 cases reported in 2006 [1]. Those 11 cases are identified by the case numbers assigned to them in the 2006 publication in the second column of this table

Case no.	Case no. in 2006 series	Age at 1st detection (year)	Age at 1st OOS exam (year)	Sex	Eye	LBD (mm)	SBD (mm)	Shape	Relevant associated ocular features	Systemic associations	Follow-up (mo)
1	1	2 months	2 months	F	R	28.0	15.5	Circular-ovoid	None	None	73.9
2	2	8 months	8 months	F	L	15.5	13.5	Circular-ovoid	None	None	176.4
3	-	2	2	M	L	6.0	5.0	Circular-ovoid	None	None	-
4	-	2	2	M	L	18.0	14.0	Circular-ovoid	RB in fellow eye	None	25.3
5	-	4	4	F	R	6.0	4.5	Circular-ovoid	None	None	4.9
6	-	5	7	F	R	37.0	23.5	Irregular-geographic	None	None	20.1
7	-	8	8	M	L	8.5	6.5	Irregular-geographic	None	None	74.9
8	3	8	8	F	R	16.0	10.5	Circular-ovoid	None	None	-
9	-	8	8	F	L	9.0	7.5	Circular-ovoid	None	Linear sebaceous nevus	99.6
10	-	9	9	F	R	26.0	18.0	Irregular-geographic	None	None	6.4
11	-	9	10	F	R	14.5	11.0	Irregular-geographic	None	None	14.8
12	-	11	11	F	R	13.0	9.0	Irregular-geographic	None	None	45.3
13	-	12	12	F	L	8.5	6.5	Irregular-geographic	None	None	0.9
14	-	12	12	M	R	10.0	9.0	Circular-ovoid	None	None	9.6
15	4	14	14	F	R	20.0	13.5	Circular-ovoid	None	None	26.4
16	-	14	15	M	L	28.0	21.0	Irregular-geographic	None	None	13.1
17	5	12	17	F	R	22.5	20.5	Irregular-geographic	None	Café au lait spot	-
18	6	17	17	M	L	13.0	9.5	Irregular-geographic	None	None	1.0
19	-	6	18	F	R	15.5	15.0	Circular-ovoid	None	None	141.9
20	-	23	24	F	L	13.5	10.0	Circular-ovoid	None	None	12.1
21	-	25	29	F	L	10.0	9.0	Circular-ovoid	CN in fellow eye; FANNUMs both eyes	None	166.8
22	7	33	33	M	R	13.0	8.5	Irregular-geographic	None	None	24.1
23	-	36	38	F	L	9.5	8.5	Circular-ovoid	None	None	24.1
24	-	18	40	M	L	19.0	9.5	Irregular-geographic	sIUM IR & CH fellow eye; FANNUM ipsi.	Congenital deafness	-
25	-	41	41	M	R	9.5	9.0	Circular-ovoid	CMM arising in ICM	None	182.1
26	8	28	41	M	L	9.0	7.0	Circular-ovoid	None	None	-
27	9	43	43	F	L	8.5	7.5	Circular-ovoid	FANNUM ipsi.	None	0.6
28	-	45	46	M	R	17.0	8.5	Irregular-geographic	CMM in fellow eye	None	39.9
29	-	41	50	F	R	32.0	18.0	Irregular-geographic	NONE	None	105.1
30	-	58	58	M	L	9.5	5.5	Irregular-geographic	NONE	None	36.2
31	-	64	64	M	L	16.0	13.5	Irregular-geographic	CMM arising in ICM	None	28.8
32	10	70	70	F	L	6.0	5.0	Circular-ovoid	NONE	None	-
33	-	71	71	F	R	5.5	4.5	Circular-ovoid	NONE	None	175.6
34	-	74	74	M	R	15.0	10.5	Circular-ovoid	CN in fellow eye	None	12.1
35	-	84	84	F	L	6.0	5.5	Circular-ovoid	AMD dry in both eyes	None	0.2
36	11	82	86	M	L	6.5	6.0	Circular-ovoid	FANNUM ipsi.	None	-
37	-	86	87	F	R	21.0	18.0	Irregular-geographic	CMM arising in ICM	None	25.4

Legend for terms, acronyms, and abbreviations that appear in table (alphabetical order): *AMD*, age-related macular degeneration; *CH*, choroid; *CMM*, choroidal malignant melanoma; *CN*, choroidal nevus; *F*, female; *FANNUMs*, focal aggregates of normal or near-normal uveal melanocytes in choroid [20]; *ICM*, isolated choroidal melanocytosis; *ipsi*., ipsilateral (i.e., involving same eye as isolated choroidal melanocytosis); *IR*, iris; *L*, left; *LBD*, largest basal diameter (arc length) of melanocytic choroidal lesion, estimated cartographically to nearest 0.5 mm; *M*, male; *mm*, millimeters; *mo*, months; *no.*, number; *OOS*, ocular oncology service (private ophthalmology practice of authors JJA and ZMC); *R*, right; *RB*, retinoblastoma; *sIUM*, sectoral isolated uveal melanocytosis; *SBD*, smallest basal diameter (arc length) of melanocytic choroidal lesion, estimated cartographically to nearest 0.5 mm; *shape*, shape of melanocytic choroidal lesion, categorized either as circular-ovoid or irregular-geographic

Fifteen patients (40.5%) were male and 22 (59.5%) were female. The isolated choroidal melanocytosis involved the right eye in 18 (48.6%) and the left eye in 19 (51.4%). No patient in this series exhibited bilateral isolated choroidal melanocytosis. However, one patient with isolated choroidal melanocytosis in one eye had combined sectoral iridic and choroidal melanocytosis (without any anterior scleral melanocytosis) in the fellow eye (case 24 in Table 1).

The 37 patients in this series ranged in age at initial evaluation in the Ocular Oncology Service from 2 weeks (a child whose melanotic fundus lesion was identified during a retinopathy of prematurity screening evaluation) to 87 years (mean age 31.5 years, median age 18 years). Eleven of the patients (29.7%) were 10 years of age or younger, 8 (21.6%) were between 11 and 21 years of age, 9 (24.3%) were 50 years of age or older, and 3 (8.1%) were 75 years of age or older when they were first evaluated in the Ocular Oncology Service. Twenty-three of these patients were referred to the Ocular Oncology Service shortly after the melanocytic choroidal lesion or other fundus abnormalities were first noted by an eye care professional. However, the melanotic choroidal lesion had been detected at a prior examination by an eye care professional and followed as a large choroidal nevus in 14 cases (cases 6, 11, 16, 17, 19, 20, 21, 23, 24, 26, 28, 29, 36, and 37 in Table 1) from 1 to 22 years (mean 5.7 years, median 3.1 years) prior to the patient’s first evaluation in the Ocular Oncology Service and diagnosis of isolated choroidal melanocytosis.

Best corrected visual acuity in the affected eye at initial evaluation in the Ocular Oncology Service ranged from 20/15 to 20/200 (median 20/20−). Visual acuity was 20/25 or better in 30 of the 37 eyes (81.1%). In contrast, visual acuity was 20/100 or worse in two eyes, one because of dry age-related macular degeneration (case 35 in Table 1) and one because of a choroidal malignant melanoma and associated secondary serous subretinal fluid (case 25 in Table 1).

**Fig. 1:**
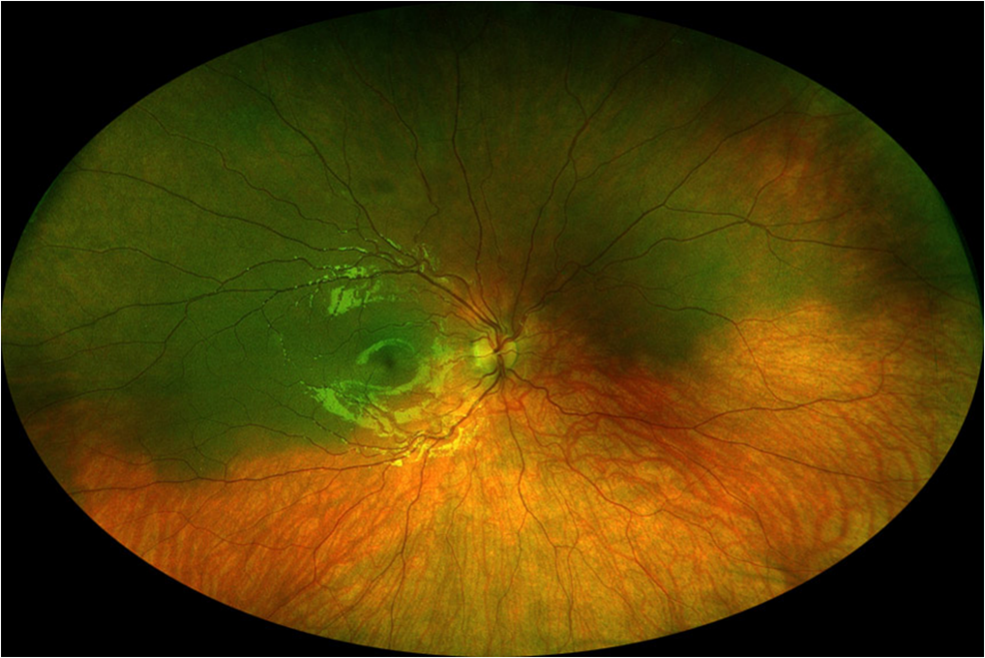
Wide-angle fundus photograph showing right fundus of a patient with isolated choroidal melanocytosis involving most of the superior hemisphere of the fundus (case 6 in Table 1)

The melanotic choroidal lesion measured from 5.5 to 37 mm in arc length largest basal diameter (mean 14.6 mm, median 13 mm) and from 4.5 to 23.5 mm in arc length smallest basal diameter (mean 10.7 mm, median 9 mm). The largest lesion in this series (case 6 in Table 1) occupied almost the entire superior hemisphere of the fundus (Fig. 1). The lesion was 10 mm or less in arc length largest basal diameter in 16 cases (43.2%), between 10 and 15 mm in 6 (16.2%), between 15 and 20 mm in 8 (21.6%), and over 20 mm in 7 (18.9%).

**Fig. 2:**
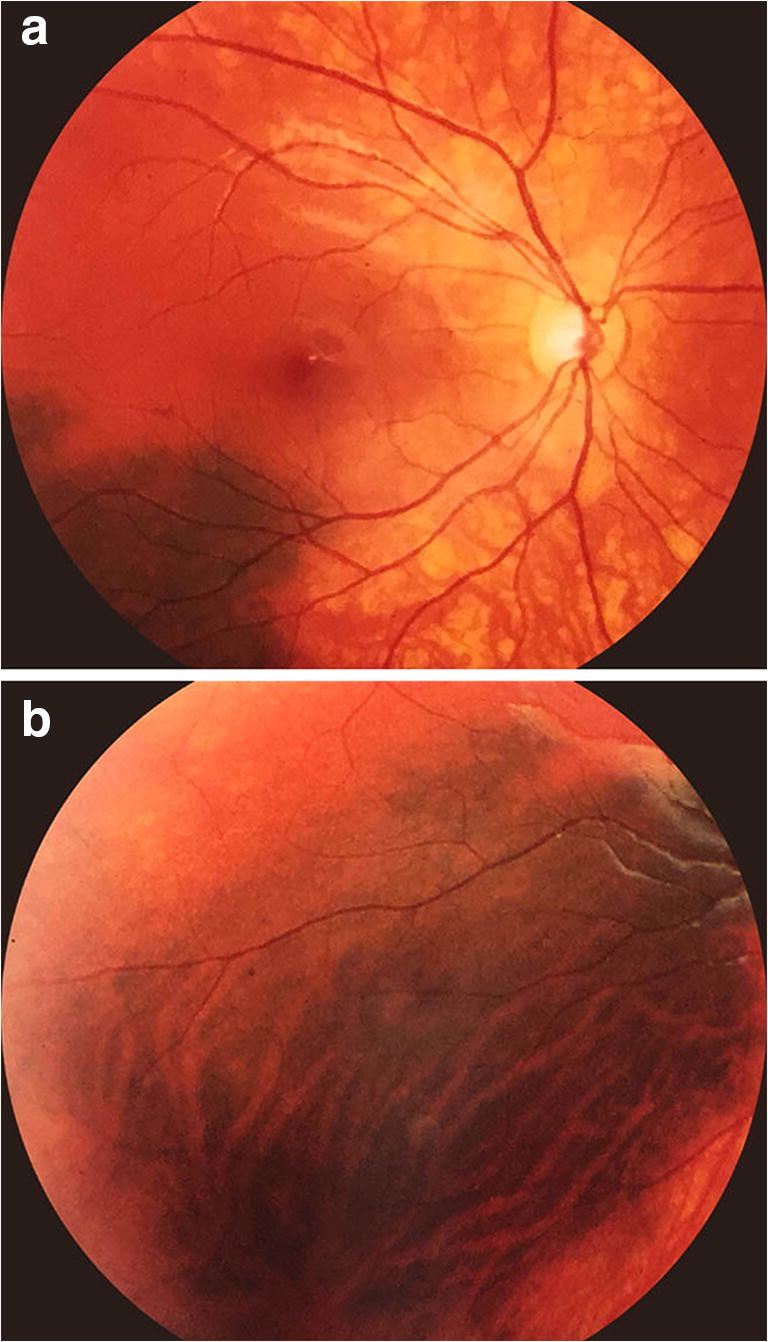
Fundus photos showing isolated choroidal melanocytosis involving macular region (**a**) and inferior midzone region (**b**) of right eye (case 8 in Table 1). Note visible large choroidal blood vessels passing through this lesion

Twenty-one of the melanotic choroidal lesions (56.8%) were roughly circular to ovoid in shape, while 16 (43.2%) were irregular or geographic in shape. Thirteen of the lesions (35.1%) had smooth margins, 20 (54.1%) had predominantly smooth margins but at least one striated-feathered margin, and 4 (10.8%) had predominantly striated margins. Large-caliber choroidal blood vessels were visible passing through the lesion (Fig. 2) in 9 cases (24.3%). None of the isolated choroidal melanocytosis lesions in this series exhibited clumps of orange pigment or disruption-clumping of the retinal pigment epithelium overlying the flat lesion or associated serous subretinal fluid attributable to the lesion; however, three patients who were found to have a choroidal malignant melanoma associated with the patch of isolated choroidal melanocytosis (cases 25, 31, and 37 in Table 1) all exhibited clumps of orange pigment and serous subretinal fluid associated with the nodular choroidal tumor. One patient (case 29 in Table 1) exhibited a multitude of drusen on the surface of the lesion both inferonasally and superonasally from the optic disc; however, this patient also had multiple similar-appearing drusen in the corresponding fundus locations in the contralateral eye. Consequently, we did not attribute these drusen to the flat melanocytic choroidal lesion.

The choroidal lesion extended beneath the fovea in 18 cases (48.6%) and to the optic disc in five (13.5%). Four of the lesions that extended to the optic disc surrounded more than half the circumference of the disc. Three patients in this series had a lesion that was both subfoveal and extended to the optic disc. Twelve of the lesions (32.4%) were extrafoveal and extrapapillary but located within one disc-foveal distance (approximately 3 to 3.5 mm) from either the fovea or optic disc or both. Five of the lesions (13.5%) were located more than one disc-foveal distance from both the optic disc and fovea. Twenty-seven of the lesions (73.0%) were located entirely posterior to the ocular equator while ten (27.0%) straddled the equator. Three of these latter lesions extended just anterior to the ora serrata, but none of the lesions extended to the pars plicata. The center point of the isolated choroidal melanocytosis patch was located posterior to the ocular equator in all 37 cases. The center point location of the lesion was categorized as posterior (indicating that it was located within one disc-foveal distance from the optic disc or fovea or both) in 6 cases, superotemporal in 5, temporal in 9, inferotemporal in 9, inferior in 2, inferonasal in 2, nasal in 1, superonasal in 1, and superior in 2.

Associated ocular features in the 37 cases (Table 1) included viable unilateral non-familial retinoblastoma in the fellow eye in one case (case 4 in Table 1 [21]), a classic choroidal nevus contralateral to the isolated choroidal melanocytosis in 2 cases (cases 21, and 34 in Table 1), one or more focal aggregates of normal or near-normal uveal melanocytes (FANNUMs [20]) of the choroid ipsilateral to the isolated choroidal melanocytosis in 4 cases (cases 21, 24, 27, and 36 in Table 1), and one or more FANNUMs of the choroid contralateral to the isolated choroidal melanocytosis in 1 case (case 21 in Table 1).

**Fig. 3:**
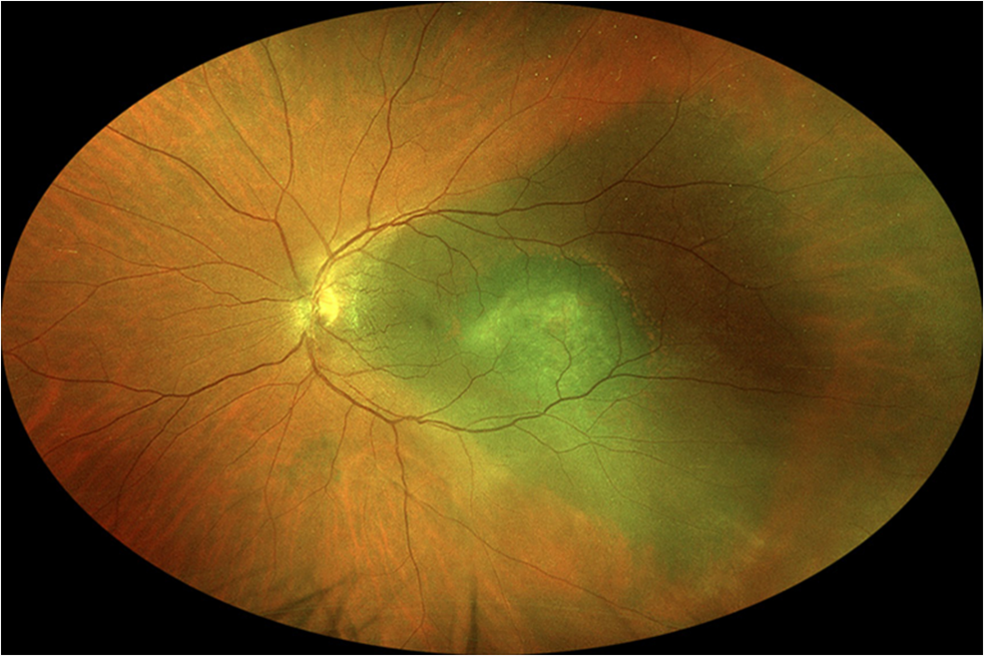
Fundus photo showing small choroidal malignant melanoma centered within triangular patch of isolated choroidal melanocytosis (case 31 in Table 1). Note striate anterior margins of the patch of choroidal melanocytosis

Four patients in this series were noted to have a choroidal malignant melanoma at initial evaluation in the Ocular Oncology Service. In one of these patients (case 28 in Table 1), the choroidal melanoma (14 mm × 8.5 mm in basal diameters × 5.5 mm in thickness) was contralateral to the isolated choroidal melanocytosis. In the other three patients (cases 25, 31, and 37 in Table 1), the choroidal melanoma was associated with the patch of isolated choroidal melanocytosis and appeared to have arisen from that patch. In case 25, the nodular tumor measured approximately 7 mm × 5.5 mm in basal diameters × 1.8 mm in maximal thickness and was localized to the foveal aspect of the isolated choroidal melanocytosis. In case 31 (Fig. 3), the nodular choroidal tumor measured 8 mm × 7 mm in basal diameters × 2.0 mm in maximal thickness and was just inferior-posterior to the center point of the isolated choroidal melanocytosis. In case 37, the nodular choroidal tumor measured 12.5 mm × 10 mm in basal diameters × 2.0 mm in thickness and was localized to the inferior aspect of the isolated choroidal melanocytosis. As mentioned above, all three of these tumors were associated with prominent clumps of overlying orange pigment and shallow overlying and inferiorly dependent serous subretinal fluid. All four of the choroidal malignant melanomas in this series (i.e., the three associated with the isolated choroidal melanocytosis and the one contralateral to the eye with isolated choroidal melanocytosis) were managed by I-125 plaque radiotherapy. In the three cases of choroidal melanoma associated with isolated choroidal melanocytosis, the location of the tumor margins was confirmed intraoperatively by indirect ophthalmoscopy with scleral depression because ocular transillumination did not distinguish between the tumor and the choroidal melanocytosis. In contrast, the choroidal melanoma not associated with choroidal melanocytosis was localized entirely by intraoperative ocular transillumination. The three choroidal tumors associated with choroidal melanocytosis were all treated with a radioactive plaque that was 4 to 5 mm larger in effective diameter than the preoperatively estimated largest basal diameter of the tumor. The plaque in these cases provided a “safety zone” approximately 2 to 2.5 mm wide or wider around the nodular tumor. No attempt was made to irradiate the entire patch of choroidal melanocytosis to a therapeutic dose in these cases. In contrast, the choroidal melanoma contralateral to the eye with isolated choroidal melanocytosis in our series was treated with a radioactive plaque that was only 2 mm larger in effective diameter than the preoperatively estimated largest basal tumor diameter. This plaque provided a “safety zone” that was only about 1 mm wide around the tumor.

Seven patients in this series were evaluated in the Ocular Oncology Service on one occasion only and advised to follow-up at least annually with their referring ophthalmologist. Four patients were reevaluated within 1 month following initial evaluation in the Ocular Oncology Service to assure us and them that the choroidal lesion was not expanding rapidly. The melanocytic choroidal lesion in these 4 patients appeared unchanged in size, so these patients were also referred back to their primary ophthalmologist for follow-up. The remaining 26 patients in this series were all followed for 3 months or longer (mean 5.0 years, median 2.3 years, minimum 4.9 months, maximum 15.2 years). None of the patches of isolated choroidal melanocytosis in these 26 patients, including those of the three patients with a plaque-irradiated choroidal melanoma associated with the choroidal melanocytosis, showed any appreciable expansion of the flat melanotic choroidal lesion. The four plaque-irradiated cases all showed satisfactory partial post-irradiation flattening of the nodular choroidal melanoma that was sustained through available follow-up. Unfortunately, the patient with choroidal melanoma contralateral to the eye with isolated choroidal melanocytosis (case 28 in Table 1) developed metastasis from his choroidal melanoma and died of that metastasis 3.3 years following treatment. The other three patients with choroidal melanoma remain metastasis-free through follow-up of 25, 28, and 182 months.

## Discussion

### Complete and partial forms of ocular melanocytosis

Congenital ocular melanocytosis has long been recognized as a distinct clinical and pathological entity [10]. The principal ocular abnormalities in this condition are congenital diffuse melanotic hyperpigmentation of the uvea and ipsilateral patchy congenital scleral melanotic pigmentation. In complete generalized ocular melanocytosis, the iris in the affected eye is much darker than that of the unaffected fellow eye and the fundus red reflex of the affected eye is substantially darker in color than that of the fellow eye. The uvea in such eyes contains plump polyhedral melanocytes that are densely packed with melanin granules [22]. Although the affected iris and choroid are frequently thickened slightly compared with these layers in a comparable region of the unaffected fellow eye [22], this slight thickening is not usually evident by ophthalmoscopy or conventional ocular ultrasonography; however, slight thickening of the involved choroid in ocular melanocytosis has been documented by optical coherence tomography [23]. When coupled with ipsilateral cephalic cutaneous melanotic hyperpigmentation, this disorder is referred to as oculodermal melanocytosis [24]. While usually a unilateral disorder, bilateral cases have been reported [25]. Congenital ocular melanocytosis has been identified as an important risk factor for development of primary uveal malignant melanoma [26].

Partial forms of congenital ocular melanocytosis, including sector ocular melanocytosis [9], isolated iris melanocytosis [27], and isolated choroidal melanocytosis [1], have all been reported. In sector ocular melanocytosis, the iris, choroid, and sclera are generally involved in a limited portion (sector) of the affected eye. In isolated iris melanocytosis, the iris is abnormally hypermelanotic in whole or in part, but scleral and choroidal melanocytosis are absent. In isolated choroidal melanocytosis, a portion (sector, patch) of the choroid is congenitally hypermelanotic, while scleral and iridic melanocytosis are absent. The extent of the choroidal melanocytosis in such cases ranges from a small patch no more than a few millimeters in diameter to involvement of more than half the fundus. The overlying retina appears unremarkable ophthalmoscopically and by optical coherence tomography. Just as with complete ocular melanocytosis, most affected persons exhibit unilateral involvement; however, bilateral cases categorized as isolated choroidal melanocytosis have been reported [3–8]. Partial (sectoral) forms of complete ocular melanocytosis have been associated with an increased risk of primary uveal melanoma arising from the originally flat melanocytic choroidal lesion [9]; however, the risk in such eyes appears to be much lower than that associated with complete generalized ocular melanocytosis.

### Choroidal malignant melanoma associated with isolated choroidal melanocytosis

In our initial description of isolated choroidal melanocytosis, we speculated that the melanotic choroidal patch in affected eyes might also be associated with an increased frequency of primary uveal malignant melanoma [1]; however, to our knowledge, no case of well-documented isolated choroidal melanocytosis that has spawned a choroidal malignant melanoma has been reported to date. In the current series, there were three patients who had a choroidal malignant melanoma associated with what we believe to be isolated choroidal melanocytosis. Because none of these patients had been documented to have a flat melanotic choroidal lesion consistent with isolated choroidal melanocytosis prior to detection of the choroidal malignant melanoma, we cannot prove that the melanocytic choroidal patch was present as a congenital lesion and gave rise to the choroidal malignant melanoma in any one of them. Because each of these tumors was treated by I-125 plaque radiotherapy, we also have no histopathologic confirmation of the nature of the flat melanotic choroidal lesion in these cases. The fact that the flat patch of choroidal melanotic hyperpigmentation did not expand appreciably in any of these three cases following plaque radiotherapy is at least consistent with our contention that the flat melanocytic patch in these cases was benign (but premalignant) and possibly congenital.

### Summary of descriptive features of isolated choroidal melanocytosis

The 37 persons in our series categorized as having isolated choroidal melanocytosis all had a discrete circular-ovoid or irregular-geographic area of clinically flat melanotic choroidal hyperpigmentation that was indistinguishable from patches of choroidal involvement in patients with sector ocular melanocytosis [9]. They also all had absence of ipsilateral iridic and anterior scleral melanocytosis and largest arc length basal diameter of the melanotic choroidal lesion greater than 5 mm. None of them had visual acuity impairment or visual symptoms attributed to the flat melanotic choroidal lesion. The 34 persons in our series whose patch of choroidal melanocytosis was not associated with a choroidal malignant melanoma all had no measurable thickness of the melanotic choroidal lesion by B-scan ultrasonography. In contrast, the three patients in our series who did have a choroidal malignant melanoma associated with the patch of isolated choroidal melanocytosis all had localized choroidal thickening corresponding to the malignant tumor but none corresponding to the uninvolved portions of the choroidal melanocytosis. None of the 23 lesions without associated choroidal malignant melanoma that were followed for more than 3 months in this series exhibited any appreciable basal expansion or thickening during that follow-up. In addition, none of the three patches of clinically diagnosed isolated choroidal melanocytosis associated with a choroidal malignant melanoma showed any appreciable basal expansion during follow-up after plaque radiotherapy of the nodular choroidal tumor.

### Differential diagnosis of isolated choroidal melanocytosis

The relevant differential diagnosis of isolated choroidal melanocytosis having the clinical characteristics of the cases comprising the current series includes broad-based (diffuse) choroidal nevus [19], diffuse choroidal melanocytoma [28–30], acquired bilateral patchy-streaky choroidal melanocytic fundopathy associated with systemic disorders such as cutaneous vitiligo [11, 12] and Waardenburg syndrome [13–15] but also occurring idiopathically in some individuals [5, 6], acquired bilateral zonal choroidal melanocytic fundopathy that is age-related [4, 8, 16] or associated with treatment with certain therapeutic agents [17], and diffuse uveal melanocytic proliferation associated with an underlying systemic malignancy [31].

#### Broad-based (diffuse) choroidal nevus

The lesion most likely to be confused with isolated choroidal melanocytosis is the broad-based (diffuse) choroidal nevus [19]. Tumors of this type are primary benign choroidal neoplasms composed of atypical but non-anaplastic uveal melanocytes [22]. Unlike isolated choroidal melanocytosis, diffuse choroidal nevi are rarely if ever congenital or even identified prior to the age of 20 years. Although lesions of this type can be relatively thin compared with their basal dimensions, almost all choroidal nevi that that are over 5 mm in largest basal diameter are measurably thick by B-scan ultrasonography. Diffuse choroidal nevi are also likely to exhibit surface drusen and disruption and/or clumping of the retinal pigment epithelium overlying the lesion.

#### Diffuse choroidal melanocytoma

The choroidal melanocytoma is a benign tumor of the choroid composed exclusively or in large part of non-anaplastic plump polyhedral melanocytes indistinguishable from the cells that comprise optic disc melanocytomas and involve the choroid diffusely in eyes of dark-skinned individuals and in complete generalized ocular melanocytosis [22, 28]. Choroidal melanocytomas are believed by some authors to be hamartomas due to localized hyperplasia of the component cells and are regarded as an “exuberant” form of choroidal melanocytosis [29]. As tumors, lesions of this type are all measurably thicker than the surrounding uninvolved choroid by B-scan ultrasonography. Such tumors tend to be darkly and diffusely melanotic. They are almost always unilateral and unifocal. They are much less likely than choroidal nevi to exhibit surface drusen and retinal pigment epithelial clumping.

#### Patchy-streaky choroidal melanocytic fundopathy

Another melanocytic choroidal disorder that can be mistaken for isolated choroidal melanocytosis is a condition we refer to as “patch-streaky choroidal melanocytic fundopathy”. Unlike isolated choroidal melanocytosis, this disorder is always acquired and almost always bilateral and relatively symmetric in the two eyes. The characteristic fundus features of this disorder are multiple discrete and sometimes confluent patches or streaks of melanotic choroidal pigmentation and multifocal confluent areas of choroidal hypomelanosis. In some cases, there is concurrent acquired diffuse or multifocal hypomelanosis of the iris. Most reported patients with this form of patchy-streaky choroidal melanocytic fundopathy have had Waardenburg syndrome [13–15]; however, some identical appearing cases without evidence of this syndrome (idiopathic cases) have been reported [3]. In other cases, there is no concurrent acquired iris depigmentation. Most reported patients with this form of patchy-streaky choroidal melanocytic fundopathy without iris depigmentation have been found to have cutaneous vitiligo [11, 12]; however, idiopathic forms of this form of the disorder have also been reported [5, 6]. In cases of both Waardenburg syndrome fundopathy and cutaneous vitiligo fundopathy, optical coherence tomography (OCT) studies have generally shown the melanotic patches to be normal in thickness and the hypomelanotic areas to have decreased thickness, presumably due to loss of uveal melanocytes in those areas [32, 33].

#### Zonal choroidal melanocytic fundopathy

Still another choroidal melanocytic disorder that can be mistaken for isolated choroidal melanocytosis is what we call “zonal choroidal melanocytic fundopathy”. Similar to the patchy-streaky choroidal melanocytic fundopathy described in the preceding paragraph, this disorder is also always acquired and bilateral and relatively symmetric in the two eyes. Three principal zonal patterns of this disorder have been identified. The most commonly encountered pattern is posterior choroidal melanosis and surrounding midzonal and peripheral choroidal hypomelanosis [16]. A second clinical pattern of this disorder is posterior choroidal hypomelanosis with retained peripheral choroidal melanosis [17]. A third (and least common, in our experience) pattern of acquired bilateral zonal choroidal melanocytic fundopathy is midzonal choroidal hypomelanosis with retained posterior and peripheral choroidal melanosis. Cases of the two former types have been evaluated by OCT, and the hypomelanotic areas of the choroid in such cases have been shown to be abnormally thinned relative to comparable fundus areas in unaffected persons [16, 17]; in contrast, the melanotic choroidal zones in such cases appear to be relatively normal in thickness. Abramson and coworkers have applied the term “leptochoroid” to the thinned choroidal areas in such eyes [16, 17]. They have associated the first pattern mentioned above with advanced age, and they have associated the second pattern with systemic therapy using checkpoint inhibitors. Unlike isolated choroidal melanocytosis, the patches of choroidal depigmentation tend to expand during follow-up.

#### Diffuse uveal melanocytic proliferation associated with systemic malignant neoplasm

This disorder is an acquired paraneoplastic stimulation of uveal melanocytes related to an underlying non-melanoma systemic malignant neoplasm (usually a carcinoma) [31]. The characteristic ocular features of this disorder are relatively rapid onset, bilaterality in almost all cases, diffuse proliferation of uveal melanocytes in the affected eyes, and an unusual associated pattern of giraffe or leopard spot chorioretinopathy [34]. In many cases, there is accentuated multifocal proliferation of melanocytes rather than uniform stimulation of all uveal melanocytes. The iris as well as the choroid and ciliary body stroma is thickened. In phakic patients, rapidly developing opalescent cataract in each eye is a frequent feature. Visual acuity is usually markedly impaired in a progressive fashion. In rare instances, unilateral forms of this disorder have been recognized [35, 36].

### Differential diagnosis of isolated choroidal melanocytosis associated with choroidal malignant melanoma

In addition to the disorders mentioned in the preceding section, one must consider diffuse choroidal malignant melanoma [37], nodular choroidal malignant melanoma with adjacent “horizontal” intrachoroidal proliferation of melanoma cells [37], nodular choroidal malignant melanoma arising from a broad-based choroidal nevus [19], and nodular growth of a choroidal melanocytoma [38, 39] as lesions in the differential diagnosis of choroidal malignant melanoma associated with isolated choroidal melanocytosis.

#### Diffuse choroidal malignant melanoma

The diffuse choroidal malignant melanoma is a specific subcategory of primary uveal malignant melanoma characterized by a relatively broad basal diameter of the tumor compared with its maximal thickness and tapering margins surrounding its thickest central portion [37]. The tumor is composed of anaplastic choroidal melanocytes that are more aggressive-appearing histopathologically on average than those of non-diffuse choroidal malignant melanomas. Tumors of this type are always acquired and unilateral and unifocal in the affected eye. Most persons with such a tumor are older adults. The entire tumor is likely to enlarge progressively if the patient is monitored without treatment following initial detection of the tumor.

#### Nodular choroidal malignant melanoma with adjacent “horizontal” intrachoroidal growth

Some primary choroidal malignant melanomas develop as a focal nodular tumor de novo or from a preexistent choroidal nevus and subsequently give rise to “horizontal” flat expansion into the adjacent previously normal choroid. The principal distinction between such a tumor and a choroidal melanoma arising from isolated choroidal melanocytosis is the expected progressive expansion of the flat melanocytic portion of the lesion within a relatively short period of follow-up if the patient is monitored without treatment after initial lesion detection.

#### Nodular choroidal malignant melanoma arising from a broad-based choroidal nevus

If a choroidal malignant melanoma arises from a broad-based choroidal nevus that had thin tapering margins and the combined abnormalities are not detected until adulthood, it may be almost impossible to differentiate such a lesion clinically from a choroidal melanoma arising from a patch of isolated choroidal melanocytosis; however, if such an eye is enucleated, histopathological analysis should be able to make this distinction.

#### Diffuse or nodular growth of a choroidal melanocytoma

Some benign choroidal melanocytomas form massive diffuse posterior uveal tumors or focally nodular choroidal tumors, some with invasive clinical features such as apical eruption through Bruch’s membrane [38, 39], that cannot be distinguished clinically from diffuse or focally nodular choroidal malignant melanomas. Tumors of this type are rarely suspected clinically and are usually diagnosed post-enucleation based on histomorphologic features of the tumor cells.

#### Isolated choroidal melanocytosis less than 5 mm in the largest basal diameter

Assuming that one accepts that isolated choroidal melanocytosis really exists as a distinct clinical entity, it is certainly possible that some discrete flat melanocytic choroidal lesions less than 5 mm in the largest basal diameter are also patches of isolated choroidal melanocytosis. Unfortunately, it is likely to be impossible to differentiate such lesions reliably from small choroidal nevi, small choroidal melanocytomas, and focal aggregates or normal or near-normal uveal melanocytes (FANNUMs) in the choroid [20] by currently available clinical means.

### Certainty about clinical diagnosis (in this series and in clinical practice)

When one encounters a patient with a discrete patch of melanotic choroidal hyperpigmentation that might be isolated choroidal melanocytosis, the examiner is advised to consider (1) the age of the affected individual when the lesion is first detected, (2) the size of the lesion (both its basal dimensions and thickness), (3) the fundus location of the lesion, (4) the laterality of ocular involvement, (5) other ocular features in the involved eye, (6) the patient’s medical-surgical history, and (7) the lesion’s stability versus expansion during follow-up. True patches of isolated choroidal melanocytosis are believed to be congenital. Consequently, the younger the patient is when the lesion is first detected, the more confidence one is likely to have in the clinical diagnosis of isolated choroidal melanocytosis. For a variety of reasons, patches of isolated choroidal melanocytosis are detected rarely in the neonatal period and infrequently during the first decade of life unless a comprehensive fundus examination with binocular indirect ophthalmoscopy is performed because of recognized ametropia or strabismus, following ocular injury or infection, or as screening for disorders such as retinopathy or prematurity and retinoblastoma. Patches consistent with isolated choroidal melanocytosis that are identified in the neonatal through teenage years (a period of life when acquired choroidal nevi are extremely uncommon [18, 22]) are likely to be accepted by most ophthalmologists as congenital lesions. However, identical appearing choroidal melanotic lesions in older adults are less likely to be regarded as congenital, especially if one or more eye care professionals have examined the patient on at least one occasion prior to the lesion’s initial detection. Depending on how and by whom a patient with isolated choroidal melanocytosis was examined, there is also the possibility that some cases of this disorder may have been missed by the examiner.

The larger the patch of isolated choroidal melanocytosis in an affected patient, the greater the probability of that lesion’s detection during an ophthalmic examination early in life. In our current series, we included only melanocytic choroidal lesions over 5 mm in largest basal diameter. We did so because most of the lesions in the differential diagnosis of isolated choroidal melanocytosis that are over 5 mm in the largest basal diameter are measurably thicker than the surrounding normal choroid, at least in some portion of the lesion [19], by B-scan ultrasonography.

The more posterior the patch of isolated choroidal melanocytosis in an affected patient, the greater the probability of that lesion’s detection during an ophthalmic examination early in life. Fortunately, most of the patches of isolated choroidal melanocytosis in this series extended to within 3 to 3.5 mm from the optic disc, fovea, or both. In this location, most such lesions should be detected whenever the affected patient undergoes his/her first comprehensive eye examination. However, because the fundus lesion of this disorder does not cause any visual symptoms and is not evident on external inspection of the eyes, many patients with isolated choroidal melanocytosis and good uncorrected visual acuity in both eyes are likely not to have their fundus lesion detected until adulthood.

In our current series, isolated choroidal melanocytosis was a unilateral unifocal disorder in every case. One patient in our series did have bilateral choroidal melanocytosis; however, that patient also had anterior uveal melanocytosis in one eye. Because of this finding, we did not consider this patient to have bilateral isolated choroidal melanocytosis. Several cases categorized as bilateral isolated choroidal melanocytosis have been reported [3–8]; however, based on our review of the published fundus photographs of those cases, most of them appear to us to have been either acquired patchy-streaky choroidal melanocytic fundopathy or acquired zonal choroidal melanocytic fundopathy, as described above, and not true bilateral isolated choroidal melanocytosis. None of the reported bilateral cases was identified in the neonatal or infantile period of life. In our opinion, most cases classified as bilateral isolated choroidal melanocytosis should be regarded with considerable skepticism unless the fundus lesion in each eye is classic and the lesions are detected very early in life.

In our series, there were no instances of prominent drusen or clumping of retinal pigment epithelium on the surface of the lesion, no cases with prominent clumps of orange pigment or serous subretinal fluid associated with the flat melanocytic choroidal lesion, and no instances of giraffe or leopard spot chorioretinopathy [34] in the affected eyes. Finding of any of these features should suggest a diagnosis other than isolated choroidal melanocytosis.

In patients with bilateral or otherwise atypical presumed isolated choroidal melanocytosis, one should review the patient’s medical-surgical history to identify features such as cutaneous vitiligo, Waardenburg syndrome, an active systemic neoplasm, or unusual medical therapies such as checkpoint inhibitor treatment that might be causative of the observed abnormalities.

If one encounters a choroidal melanotic lesion he/she believes might be isolated choroidal melanocytosis, one should recommend periodic reevaluation of the lesion to determine whether it is stable in size (which one would expect if the lesion is indeed a patch of congenital choroidal melanocytosis) or expanding (which would be much more consistent with an acquired choroidal melanocytic hyperpigmentation). Based on our current series, follow-up is also important to detect a choroidal melanoma that might be spawned at any time by the patch of isolated choroidal melanocytosis.

As noted above, none of the melanotic choroidal lesions in our series prompted enucleation. We therefore have no histopathology on the flat choroidal melanotic hyperpigmentation in any of our cases. Because of this, we do not know for certain whether the hypermelanotic choroidal area was due to the presence of plump polyhedral melanocytes containing prominent intracytoplasmic melanin granules similar to those in darkly pigmented races and in complete ocular melanocytosis in some, all, or any of our patients. In spite of this lack of histologic evidence, we believe that our series of cases provides reasonably strong circumstantial evidence that isolated choroidal melanocytosis really exists as a distinct clinical and histopathological entity and that choroidal melanoma can arise from patches of isolated choroidal melanocytosis. In view of our findings, we recommend at least an annual fundus monitoring of patients with clinically diagnosed isolated choroidal melanocytosis throughout life to watch for progression of the choroidal lesion and emergence of a choroidal melanoma from that lesion.

## Data Availability

De-identified data may be evaluated upon written request.
